# Patient-Reported Outcome Measures of Psychosocial Quality of Life in Oropharyngeal Cancer Patients: A Scoping Review

**DOI:** 10.3390/jcm12062122

**Published:** 2023-03-08

**Authors:** Jennifer A. Silver, Russell Schwartz, Catherine F. Roy, Nader Sadeghi, Melissa Henry

**Affiliations:** 1 Department of Otolaryngology-Head and Neck Surgery, McGill University, 3755 Côte St. Catherine Road, Pavilion E Room E-903, Montreal, QC H3T 1E2, Canada; 2 Research Institute of the McGill University Health Centre, Montreal, QC H4A 3J1, Canada; 3 Gerald Bronfman Department of Oncology, Faculty of Medicine and Health Sciences, McGill University, Montreal, QC H3G 2M1, Canada; 4 Lady-Davis Institute for Medical Research, Jewish General Hospital, Montreal, QC H3T 1E2, Canada; 5 Segal Cancer Centre, Jewish General Hospital, Montreal, QC H3T 1E2, Canada

**Keywords:** oropharyngeal cancer, human papillomavirus, psychosocial, quality of life

## Abstract

**Background:** Oropharyngeal squamous cell carcinoma (OPSCC) patients are burdened by the effect of the disease process and treatment toxicities on organs important in everyday activities, such as breathing, speaking, eating, and drinking. There is a rise in OPSCC due to human papilloma virus (HPV)-associated OPSCC, affecting younger and healthier patients and with a better overall prognosis. Emphasis must be shared between oncologic outcomes and the effects on quality of life. While there have been efforts to study global and physical quality of life, the impact on psychosocial quality of life has not yet been specifically reviewed. **Methods:** A scoping review methodology was employed to explore the emotional, social, and mental quality of life in OPSCC patients and determine the impact of HPV status or treatment modalities. **Results:** Eighty-seven full-text articles were evaluated for eligibility. Fifteen articles met final inclusion criteria. The majority of the studies were conducted in the United States (n = 10) and study methodology was divided between cross-sectional (n = 6), prospective (n = 5), and retrospective studies (n = 4). Four psychosocial quality of life themes were explored: the impact on mental health and emotional wellbeing, social wellbeing and function, stress, and relationship and sexual behavior. Eighteen different patient-reported outcome measures were used, including both general head and neck oncology questionnaires and symptom-specific surveys. **Conclusion:** There is a paucity of research regarding the effect of OPSCC on patients’ psychosocial quality of life. Learning more about this component of quality of life can guide outreach programs and multidisciplinary involvement in improving patient care.

## 1. Introduction

While the number of head and neck cancer diagnoses is decreasing, the prevalence of oropharyngeal squamous cell carcinoma (OPSCC) has been increasing in North America due to human papillomavirus (HPV)-associated disease [1]. Currently, approximately 60–70% of OPSCC is associated with HPV, in contrast to traditional tobacco- and alcohol-related OPSCC [1,2,3,4].

HPV-associated OPSCC differs significantly from conventional OPSCC. Clinically, these patients are younger and healthier at baseline, with little or no tobacco exposure, and the prognosis is favorable with standard treatments [3,4,5]. As such, psychosocial issues related to head and neck cancer survivorship are increasingly apparent in this patient population, yet remain understudied in the scientific literature [6].

Oropharyngeal cancers originate at keystone areas for breathing, eating, and speech [7]. Patients with oropharyngeal cancer experience stress from facing their cancer diagnosis and intensive treatment regimens, with the added effects on organs essential to the activities of daily living and communication [7,8,9,10]. With improved prognosis for most OPSCC cases, goals are shared between maintaining the excellent overall survival and disease-free survival, and quality of life (QOL) [11]. Recent reviews have summarized ongoing or recently completed clinical trials attempting to de-escalate standard therapies for HPV-associated OPSCC patients to minimize or lessen treatment-related side effects [12,13].

Most reviews assessing health-related QOL in OPSCC patients focus on xerostomia, dysphagia, mastication, and other physical complaints [9,14]. While these are important markers for assessment of post-treatment toxicity, QOL is multifactorial. The World Health Organization defines QOL as “an individual’s perception of their position in life, in the context of culture and value system in their life and in relation to their goals, expectations, standards and concerns” [15]. Our definition of QOL should extend beyond physical and functional dimensions and incorporate social and emotional factors. This is especially important in head and neck cancer patients, a population in which the prevalence of diagnosed major depressive disorders is as high as 40% [16]. Head and neck cancer patients are more likely to commit suicide when compared to the general population or to patients diagnosed with 19 other cancers [17,18]. However, the changed demographic and better prognosis of HPV-associated OPSCC patients may lead to a different impact on psychosocial QOL.

There has not yet been a review addressing the psychosocial impact of oropharyngeal cancer on patients. Most research focuses on survival and on physical aspects of the disease, in striking contrast with patient-centered concerns. The primary objective of this review is to assess the broad psychosocial QOL in oropharyngeal cancer patients using patient-reported outcome measures (PROMs). Secondary objectives are to determine whether treatment regimens or HPV status play a role.

## 2. Methodology

This scoping review seeks to identify the current literature published in this field, examine how the research was conducted, and detail the key factors and gaps in knowledge. We followed the scoping review framework proposed by Arksey and O’Malley, expanded by Levac et al. [19,20]. Preferred Reporting Items for Systematic Reviews and Meta-Analyses (PRISMA) Extension for Scoping Reviews was followed as a complementary guideline [21].

### 2.1. Identifying the Research Question

This scoping review was developed to describe the nature, number, and scope of published research articles examining the relationship of psychosocial QOL in patients with OPSCC using validated patient-reported outcome metrics. 

### 2.2. Identifying Relevant Studies

A systematic literature search of PubMed, Embase, PsycINFO, and CINAHL was conducted of all articles published between 1946 and August 2022. Search terms included a combination of appropriate database MeSH terms, subject headings, and keywords for the concepts of oropharyngeal cancer, QOL, patient-reported outcome measures, and different emotional and mental states (Supplementary S1). The search strategy was developed with the assistance of a medical librarian guided by the Joanna Briggs Institute; inclusion and exclusion criteria were determined by population type, concept, and context framework (Table 1) [22].

### 2.3. Study Selection

The titles and abstracts of all identified studies were screened by two independent reviewers (JS, RS), with a senior author available to resolve conflicts not agreed upon by discussion (MH). The abstract screening protocol was discussed among authors, and criteria defined using Rayyan, a software designed to allow multiple reviewers to independently select studies for inclusion or exclusion [23]. A pilot sample of 20 abstracts was completed to ensure that both reviewers had a common understanding of the inclusion and exclusion criteria (Table 1). All article abstracts were screened in increments of 100–200 articles to regularly check inter-rater reliability and ensure consistent results.

The full-text articles were screened by two reviewers (JS, RS) with more refined criteria (Table 1). Articles were included if they were studies published in peer-reviewed journals with a population of adult patients with OPSCC who are undergoing or completed treatment for their disease (surgery, chemotherapy and/or radiation therapy (RT)). The included studies reported on QOL with validated patient-reported outcome metrics, and at least one component of psychosocial QOL was a primary or secondary outcome of the paper. The reference lists of eligible studies were also reviewed to identify any further studies that had been missed in the electronic searches.

### 2.4. Data Extraction

From the full texts, two authors (JS and RS) extracted the following data: author(s), year of publication, study design, study location, participant characteristics, PROMs employed, psychosocial QOL concepts discussed, and important findings. The psychosocial theme content analysis was compiled using NVivo software and inter-rater reliability was calculated [24]. Risk of Bias was assessed using the National Institutes of Health Quality Assessment Tool by two independent reviewers (JS and RS) [25]. Disagreements were resolved by discussion and by consulting a senior author (MH) to resolve remaining discrepancies. Prior to submission, the search was repeated and an additional two articles were included in the analysis. New articles identified since the primary search were screened and data extraction was performed by the same reviewers jointly to ensure agreement.

### 2.5. Collation, Summarizing and Reporting the Results

The data from the included studies were collated and included study demographics, PROMs employed, and psychosocial theme (mental health and emotional wellbeing, social contact, stress, and interpersonal relationships).

## 3. Results

### 3.1. Study Population and Demographics

**Figure 1:**
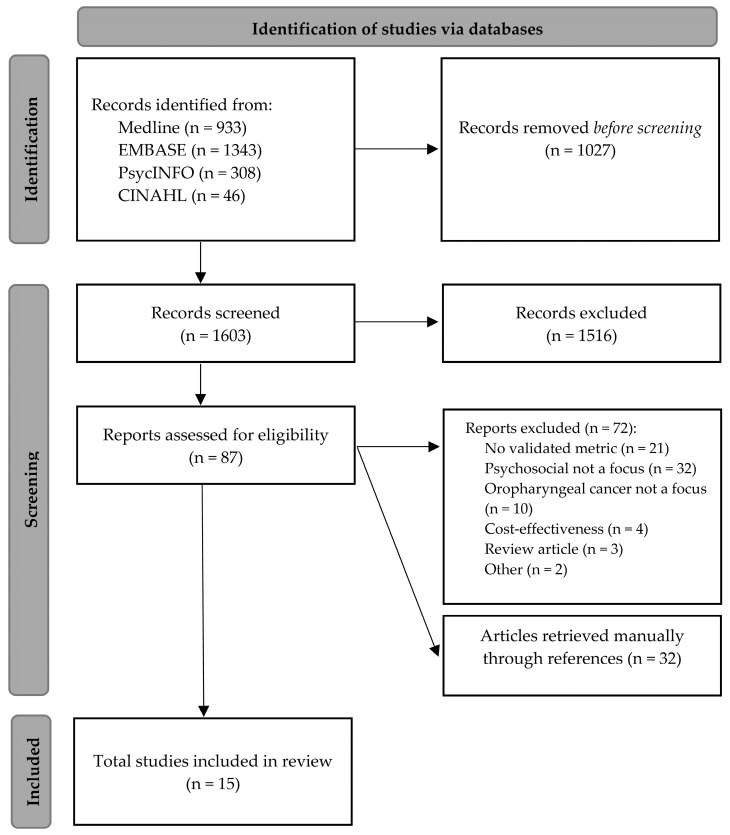
PRISMA diagram of included studies.

The study selection process is outlined in Figure 1. The databases yielded 2630 citations (Medline: 933, Embase: 1343, PsycINFO: 308, CINAHL: 39), reduced to 1603 articles after removing duplicates. Of these, 87 full-text articles were deemed to be eligible for full-text review. There was a 96.9% inter-rater reliability between the two screening authors (JS and RS). Cohen’s kappa was calculated at 0.73, representing substantial agreement [26]. Fifteen articles met eligibility criteria. Their references were screened, but no further articles met inclusion criteria. The included studies had varied study designs, cross-sectional (n = 6), prospective (n = 5), and retrospective (n = 4). The majority of the studies were conducted in the United States (n = 10), with the remainder from Australia (n = 3), and single studies from both Sweden (n = 1) and the Netherlands (n = 1). The three studies from Australia were conducted by the same research group using the same cross-sectional methodology and patient cohort. Studies were published between 2013 and 2022. Sample sizes ranged from 24 to 972 patients, with an average of 179 patients per study. The average age of participants was 59 years (range18 to 89). All studies assessed QOL post-treatment at an average of 30.9 months follow-up (range six months to six years).

### 3.2. Quality Assessment

Using the National Institutes of Health Quality Assessment Tool, the majority of the studies were rated as fair (n = 11), with the remainder graded as good (n = 3) and poor (n = 1).

### 3.3. QOL Metrics

This scoping review identified 18 different validated patient-reported outcome measures (PROMs) utilized by studies, summarized in Supplementary S2. The most frequently employed were the MD Anderson Symptom Inventory for Head and Neck cancer (MDASI-HN) (n = 5) and the European Organization for Research and Treatment of Cancer Core QOL Questionnaire (EORTC QLQ-C30) (n = 5). Within the different themes, there were symptom-specific PROMs. In the mental health category, three different depression metrics were utilized: the Center for Epidemiologic Studies Depression Scale (CES-D), Patient Health Questionnaire (PHQ), and Patient-Reported Outcomes Measurement Information System-Depression 8b (PROMIS^®^-Depression 8b). Two different anxiety metrics were used: General Anxiety Disorder-7 (GAD-7) and the Patient-Reported Outcomes Measurement Information System-Anxiety 7a (PROMIS^®^-Anxiety 7a). The variability of components of stress required symptom PROMs for each specific element (i.e., Fear of Cancer Recurrence Inventory, Decision Regret Scale).

### 3.4. Identification of Psychosocial QOL Themes and Thematic Analysis in Oropharyngeal Cancer Patients

The eligible studies reported on four main themes within psychosocial QOL (Table 2), subdivided as follows: mental health and emotional wellbeing (n = 10), social wellbeing and function (n = 4), stress (n = 5), and relationship and sexual behavior (n = 3). The content analysis of psychosocial QOL themes conducted by both reviewers yielded a Cohen’s kappa correlation coefficient of 0.77, demonstrating substantial agreement.

#### 3.4.1. Mental Health and Emotional Wellbeing

The mental health domain comprised studies addressing impacts on depression, anxiety, mood, and emotional function.

Only one study by Kaffenberger evaluated patient mental health after different treatment modalities [27]. This retrospective cohort study compared patients with advanced oropharyngeal cancer treated with primary chemoradiotherapy (CRT) (n = 44) to those treated with surgery with adjuvant RT or CRT (n = 29) and found no significant difference in depression or anxiety scores between the two cohorts, using the PHQ-8 and GAD-7 PROMs, respectively.

The studies that evaluated mental health at different time points noted improvement in mental health scores over time. Janz’s prospective cohort study exploring differences between HPV-associated OPSCC patients (n = 21) and HPV-negative oral cavity cancer patients who smoke (n = 17) found that, at 12 months, the HPV-associated OPSCC cohort had an improved depression score on the CES-D [28]. Rajeev-Kumar conducted a retrospective analysis of OPSCC patients treated with RT (n = 69) using the University of Washington QOL (UW-QOL) questionnaire and noted that anxiety and mood scores improved at 12 months compared to pre-treatment values [29].

Berg performed a cross-sectional study comparing BOT cancer patients (n = 190) to tonsillar cancer patients (n = 405) and to the general population (n = 190) [30]. This research identified better emotional function in the patients with HPV-associated OSPCC than in the HPV-negative patients on the EORTC QLQ-C30. Qualliotine’s retrospective review of OPSCC patients (n = 69) noted that a lower proportion of HPV-associated OPSCC patients use anti-depressants [31]. Korsten prospectively compared HPV-positive and HPV-negative OPSCC patients and identified greater post-treatment emotional function in the former group using the EORTC QLQ-C30 and the EORTC Head and Neck Cancer module (EORTC QLQ-HN35) [32]. Lee found decreased anxiety (*p* = 0.005) but no significant difference in mood (*p* = 0.288), using the UW-QOL scale in 25 HPV-associated OPSCC patients treated with neoadjuvant chemotherapy and transoral robotic surgery (n = 25) compared to a normative cohort [33].

Several other studies did not associate worsening mental health with HPV status (Qualliotine on CES-D initial screen, Rajeev-Kumar on UW-QOL, and Shinn using both the PHQ-9 and the CES-D) [29,31,34]. Shinn performed a prospective cohort study on 130 patients with OPSCC [34]. Casswell et al. did not compare their data of their HPV-associated cohort to HPV-negative patients [27,35,36]. Casswell and McDowell studied the same 136-patient, HPV-positive OPSCC cohort treated with CRT in their cross-sectional studies, using the Patient-Reported Outcomes Measurement Information System (PROMIS^®^) Anxiety and Depression questionnaires [35,36,37].

#### 3.4.2. Social Wellbeing and Function

The social wellbeing and function domain comprised studies addressing impacts on social quality of life, social contact, and social eating.

Berg did not identify any significant difference in social domain of the EORTC QLQ-C30 or in the EORTC QLQ-HN35 scores in BOT OPSCC patients who underwent different treatment modalities [30]. Kaffenberger did not identify differences in UW-QOL social scores when comparing CRT to surgery with adjuvant RT or CRT [27]. However, this study did establish that the mean dose of RT delivered to the ipsilateral parotid gland correlated with worse social scores. Dziegielewski performed a prospective cohort study exploring swallowing, speech, and QOL outcomes after transoral robotic surgery in 81 patients with OPSCC, using the Head and Neck Cancer Inventory (HNCI) [38]. The social QOL domain declined immediately after surgery, reaching a nadir at three months; however, this domain recovered and was similar to baseline results at one-year post-therapy.

Comparing HPV-positive and HPV-negative patients, Korsten identified better social functioning at baseline, which worsened to a greater extent during treatment, and recovered better and more quickly at follow-up compared to patients with an HPV-negative cancer [32]. However, mixed-model analysis did not demonstrate a significant difference between HPV-positive and negative patients on social contact and social eating domains. There was no difference in social scores in HPV-positive and HPV-negative patients in the two studies that performed this comparison (Berg, Dziegielewski) [30,38]. 

#### 3.4.3. Stress

Stress was a diverse theme within this scoping review, with five studies discussing four stress-related concepts: fear of cancer recurrence [35,39], overall attitude/bother or satisfaction with function [38], decisional regret [39,40], and cancer worry [28]. 

Casswell employed the Fear of Cancer Recurrence Inventory and found that this fear was present in over half of the patients, with younger patients more likely to report this stress [35]. Fear of cancer recurrence was also associated with lower global QOL, higher symptom interference with daily activities, and greater anxiety and depression scores [39]. This study used a patient perspective questionnaire, a measure developed by the researchers based on previously validated metrics.

Dziegielewski identified a significant difference in change in overall attitude in the Head and Neck Cancer Inventory (HNCI; a measure capturing patients’ ratings of their function and how much they are bothered by that function) in patients who received adjuvant RT (*p* = 0.003) and those receiving adjuvant CRT (*p* = 0.04) compared to those without adjuvant treatment [38]. There was no difference in overall attitude in HPV-positive or HPV-negative patients (*p* = 0.56).

The study by Janz used the Assessment of Survivor Concerns instrument to compare cancer worry in HPV+ OPSCC patients with smoking oral cavity cancer patients and found that there was no statistically significant difference in cancer worry score (*p* = 0.1) [28]. Cancer worry also decreased over time in both cohorts but was not statistically significant (HPV+ OPSCC: 21 to 16, *p* = 0.11, oral cavity: 16 to 15, *p* = 0.07).

Goepfert and Shaverdian both examined decisional regret in their cohorts using the Decision Regret Scale [39,40]. Goepfert’s cross-sectional study reported an average score correlating to mild decision regret (n = 935, median follow-up 6 years) [40]. A total of 15.5% of the patients did exhibit moderate to strong regret, which was significantly associated with higher T classification, combination treatment (surgery and RT/CRT), smoking at diagnosis, and high MDASI-HN symptom score (associated with dysphagia symptom). Shaverdian [39] performed a single-arm cross-sectional study of HPV-associated OPSCC patients (n = 24) enrolled in a de-escalation clinical trial protocol (induction chemotherapy and then concurrent CRT with reduced dose RT of either 54 Gy or 60 Gy based on response). Patients were satisfied overall with their treatment, agreeing that they had made the right decision to pursue a de-escalated treatment. No patient regretted the choice or was dissatisfied with their treatment at a median follow-up of 24 months.

**Table 2:** Thematic analysis of psychosocial quality of life measures in oropharyngeal cancer patients.

Primary Author, Year	Study Design	Country	Participant Characteristics	Comparator	HPV/P16 Status of Participants	Cancer Stage	Treatment	Time Period	PROM	Summary of Results
**Mental health and emotional wellbeing**										
Berg, 2021 [30]	Cross-sectional	Sweden	190 patients with BOT cancer, aged 33–84 (median 63), 137 male, 53 female	Patients with tonsillar cancer, general population	Positive: 131 Negative: 20 Missing: 39	Stage I-II: 27 Stage III-IV: 162 Missing: 1 (AJCC 7th edition)	RT: 56 CRT: 85 Surgery ± RT: 34 Surgery + CRT: 14 No adequate treatment: 1	15 months post-treatment	EORTC QLQ-C30, EORTC QLQ-H&N35	Emotional function is higher in general population and in males, worse in HPV negative patients, same in tonsil cancer patients.
Casswell, 2021 [35]	Cross-sectional	Australia	136 patients with HPV-associated oropharyngeal cancer, aged 42–87 (median 61), 114 male, 22 female	N/A	Positive: 136/136	Stage I: 74 Stage II: 22 Stage III: 40 (AJCC 8th edition)	RT: 16 CRT: 120 Salvage surgery: 1	Mean 2.8 years post-treatment (range 1–5.5 years)	EORTC QLQ-C30, MDASI-HN, PROMIS, Fear of Cancer Recurrence Inventory	Moderate levels of anxiety and depression were reported in 11% and 4% of patients, respectively. Severe levels of anxiety and depression were both reported in 1% of patients, respectively. PROMIS anxiety and depression scores were significantly associated with fear of cancer recurrence scores.
Janz, 2019 [28]	Prospective	USA	21 patients with HPV-associated oropharyngeal cancer, aged 49–76 (mean 58.2), 19 male, 2 female	17 patients with oral cavity cancer who smoke aged 32–76 (mean 55), 9 male, 8 female	Oropharynx cohort—Positive: 21/21 Oral cavity cohort—Positive 0/17	Stage IV (oropharynx): 16 Stage IV (oral cavity): 11 (AJCC 7th edition)	Surgery: 13 RT: 16 Chemotx: 17 **Combination therapies:** Surgery + RT:2 CRT: 5 Surgery + CRT: 9 Other: 3	12 month follow-up	Cancer worry “Assessment of Survivor Concerns” instrument, CES-D, Cancer Behavior Inventory	At baseline: there was no difference in depression score between HPV positive OPSCC patients and smoking oral cavity patients (*p* = 0.041) At 12-months: depression decreased over time for the HPV positive cohort (*p* = 0.03)
Kaffenberger, 2021 [27]	Retrospective	USA	44 patients with advanced oropharyngeal cancer treated with curative intent treated with primary CRT, with a mean age of 57.6, 37 male, 7 female	29 patients with advanced oropharyngeal cancer treated with curative intent treated with surgery and adjuvant RT/CRT, with a mean age of 56.7, 25 male, 4 female	Positive: 66/73 Negative: 3/73 Unknown: 4/73	Stage III: 10 Stage IVa: 62 Stage IVb: 1 (AJCC 7th edition)	CRT: 44 Surgery + RT: 9 Surgery + CRT: 20	Median follow-up post treatment 29.7 months (range 6.1–133 months)	UW-QOL, PHQ-8, GAD-7, NDI, EAT-10	On PHQ-8: no significant difference in depression scores between groups (*p* = 0.71) On GAD-7: no significant difference in anxiety scores between groups (*p* = 0.77), mean dose of RT delivered to the ipsilateral parotid correlated to more anxiety symptoms.
Korsten, 2021 [32]	Prospective	The Netherlands	78 patients with HPV-associated oropharyngeal cancer, mean age 59.9, 59 male, 19 female	120 patients with HPV-negative oropharyngeal cancer, mean age 59.9, 120 male, 72 female	Positive: 78/270	Stage I: 37 Stage II: 57 Stage III: 59 Stave IV: 103 (AJCC 7th edition)	RT: 99 Surgery: 4 Combination: 89	24 months	EORTC QLQ-C30, EORTC QLQ-H&N35	Emotional functioning mean scores were equal at baseline, 6 weeks and 3 months after treatment between HPV-positive and negative cohorts (*p* = 0.039). Scores improved more in HPV-positive patients at 6, 12, and 24 months compared HPV-negative patients.
Lee, 2022 [33]	Cross-sectional	USA	25 patients with HPV-associated oropharyngeal cancer, aged 41–80 (median 58), 23 male, 2 female	N/A	Positive: 25/25	Stage II: 1 Stage III: 2 Stage IVa: 21 Stage IVb: 1 (AJCC 7th edition	All received neoadjuvant chemotherapy and transoral robotic surgery	Mean 4.3 years (2.0–7.6 years)	UW-QOL	Patients treated with this protocol reported less anxiety compared to the normative cohort, demonstrating near-normal recovery in long-term outcomes (*p* = 0.005). There was no significant difference in mood scores of trial participants compared to controls (*p* = 0.288).
McDowell, 2021 [36]	Cross-sectional	Australia	136 patients with HPV-associated oropharyngeal cancer, aged 42-87 (median 61), 114 male, 22 female	N/A	Positive: 136/136	Stage I: 74 Stage II: 22 Stage III: 40 (AJCC 8th edition)	RT: 16 CRT: 120 Salvage surgery: 1	Mean 2.8 years post-treatment (range 1–5.5 years)	EORTC QLQ-C30, MDASI-HN, PROMIS, Fear of Cancer Recurrence Inventory	Anxiety (t-score 53.5 vs. 44.1, *d* = 0.80), and depression (t-score 42.8 vs. 51.3, *d* = 0.84) scores were significantly worse in the low functioning subgroup. PROMIS anxiety score: normal/low: 88.9%, moderate: 9.6%, severe: 1.5% PROMIS depression score: normal/low: 95.6%, moderate: 3.7%, severe: 0.7%. Increasing age is associated with worse anxiety scores (−0.2/year increase, *p* = 0.034)
Qualliotine, 2017 [31]	Retrospective	USA	65 patients with oropharyngeal cancer between October 2011 and September 2014 who had completed the depression screening questionnaire prior to treatment, aged 44-88 (median 59.9), 55 male, 10 female	N/A	Positive: 50 Negative 15	Stage I or II: 4 Stage III or IV: 61 (AJCC 7th edition)	N/A	N/A	CES-D	A lower proportion of HPV-associated OPSCC patients than HPV-negative patients reported using antidepressants (8% vs. 27%, *p* = 0.05). 44.9% of the patients screened positive for depression. No association of depression score and HPV status.
Rajeev-Kumar, 2019 [29]	Retrospective	USA	69 patients treated with curative intent RT between 2013 and 2016 with up to 3 year follow-up, with a mean age of 58.3, 51 male, 18 female	N/A	Positive: 43 Negative: 26	Stage I: 4 Stage II: 7 Stage III: 12 Stage IVa: 41 Stage IVb: 4 (AJCC 7th edition)	Pre-RT surgery: 37 RT: 69 Induction chemotx: 16 Concurrent CRT 38	12 months post-RT	UW-QOL	Of the 51 patients with active alcohol use, 11.8% had a severe mood score and 33.3% had a severe anxiety score before starting RT. After 12 months, 88% of those patients returned to baseline or better mood (only 52% response). At consultation, anxiety was worse than mood score. At 12 months, anxiety remained mildly worse than mood but both were better than pre-treatment. Multivariate regression: no association between worse emotional status and patient/disease characteristics at 12 months, PEG placement, surgery versus CRT, HPV infection. Longer duration of treatment is more likely to be associated with worse mood (>50 days of treatment). Physical symptom worsening is associated with worse anxiety (taste scores, saliva scores) and with worse mood (swallow scores).
Shinn, 2016 [34]	Prospective	USA	130 patients diagnosed with new diagnosis of oropharyngeal cancer between March 2005 and June 2007 treated with RT, aged 28.4–78.5 (mean 56.8), 94 male and 108 male, 22 female	N/A	Positive: 15/22 Negative 7/22 (Only 22 patients tested)	Stage I or II: 10 Stage III or IV: 119 Missing: 1 (AJCC 7th edition)	RT: 130 Neoadjuvant chemotx: 47 Concurrent CRT: 51	Median of 4.9 years (range of 0.1–6 years)	PHQ-9, CES-D	19 patients (15%) screened positive for depression at baseline. In the univariate analysis of the PHQ-9, depression’s association with survival was borderline (*p* = 0.061) but significant in the multivariate analysis (*p* = 0.022). Dichotomized, PHQ-9 positive depression was associated with overall survival (*p* = 0.022). As a multivariate model, for every increased unit of the PHQ-9, the risk for reduced survival increased by a factor of 10%. Depression was associated with disease recurrence in univariate (*p* = 0.028) and multivariate analysis (*p* = 0.025). For every increased unit of the PHQ-9, the risk for recurrence increased by a factor of 10%. No association of HPV status and depression
**Social wellbeing and function**										
Berg, 2021 [30]	Cross-sectional	Sweden	190 patients with BOT cancer, aged 33–84 (median 63), 137 male, 53 female	Patients with tonsillar cancer, general population	Positive: 131 Negative: 20 Missing: 39	Stage I–II: 27 Stage III–IV: 162 Missing: 1 (AJCC 7th edition)	RT: 56 CRT: 85 Surgery ± RT: 34 Surgery + CRT: 14 No adequate treatment: 1	15 months post-treatment	EORTC QLQ-C30, EORTC QLQ-H&N35	Compared to the general population, BOT patients have worse social function (*p* < 0.001), social eating (*p* < 0.001), social contact (*p* < 0.001). No difference in social domains in BOT patients who are stage I-II versus III-IV, males versus females, HPV+ versus HPV-, different treatment modalities or adjuvant treatment regimens. Patients with BOT cancer had worse social eating scores than patients with tonsil cancer (*p* = 0.001).
Dziegielewski, 2013 [38]	Prospective	USA	81 patients with oropharyngeal cancer treated with transoral robotic surgery	N/A	HPV positive: 51 HPV negative: 20 p16 positive: 60 p16 negative: 11 Missing: 10	Stage I: 7 Stage III: 9 Stage IV: 63 Missing: 2 (AJCC 7th edition	Surgery: 81 Adjuvant RT: 69 Adjuvant CRT: 49	12 month post-operatively	HNCI	All health-related quality of life scores declined at 3 weeks post = operatively, including social scores, which continued to drop but reached the nadir at 3 months. Social scores recovered and were indifferent from baseline (*p* > 0.05) at 12 months. No difference of social function (*p* = 0.81) or social attitude (*p* = 0.57) when in HPV+ or HPV-patients.
Kaffenberger, 2021 [27]	Retrospective	USA	44 patients with advanced oropharyngeal cancer treated with curative intent treated with primary CRT, with a mean age of 57.6, 37 male, 7 female	29 patients with advanced oropharyngeal cancer treated with curative intent treated with surgery and adjuvant RT/CRT, with a mean age of 56.7, 25 male, 4 female	Positive: 66/73 Negative: 3/73 Unknown: 4/73	Stage III: 10 Stage IVa: 62 Stage IVb: 1 (AJCC 7th edition)	CRT: 44 Surgery + RT: 9 Surgery + CRT: 20	Median follow-up post treatment 29.7 months (range 6.1–133 months)	UW-QOL, PHQ-8, GAD-7, NDI, EAT-10	The mean dose delivered to the ipsilateral parotid gland was correlated with worse scores on the social aspects of the UWQOL No difference in social score based on treatment modality.
Korsten, 2021 [32]	Prospective	Canada	78 patients with HPV-associated oropharyngeal cancer, mean age 59.9, 59 male, 19 female	120 patients with HPV-negative oropharyngeal cancer, mean age 59.9, 120 male, 72 female	78/270	Stage I: 37 Stage II: 57 Stage III: 59 Stave IV: 103 (AJCC 7th edition)	RT: 99 Surgery: 4 Combination: 89	24 months	EORTC QLQ-C30, EORTC QLQ-H&N35	For HPV-associated patients, social functioning was better before treatment, worsened during treatment, and recovered better and faster at follow-up compared to patients with an HPV-negative cancer (*p* = 0.033). On mixed model analysis, social contact and social eating did not demonstrate a significant difference between HPV-positive and negative patients.
**Stress**										
Casswell, 2021 [35]	Cross-sectional	Australia	136 patients with HPV-associated oropharyngeal cancer, aged 42-87 (median 61), 114 male, 22 female	N/A	Positive: 136/136	Stage I: 74 Stage II: 22 Stage III: 40 (AJCC 8th edition)	RT: 16 CRT: 120 Salvage surgery: 1	Mean 2.8 years post-treatment (range 1–5.5 years)	EORTC QLQ-C30, MDASI-HN, PROMIS, Fear of Cancer Recurrence Inventory	Clinically significant fear of cancer recurrence was reported in 53% of patients (72/135). Younger patients were more likely to report high fear of cancer recurrence (−0.9/5 years; *p* = 0.031). Those with higher fear of cancer recurrence also had lower global QOL (−0.8/10 unit increase; *p* = 0.012), had higher symptom interference with daily activities (0.8/unit increase; p = 0.17) (MDASI-HN), and greater anxiety (0.4/unit; *p* < 0.001) and depression scores (0.3/unit; *p* < 0.001) (PROMIS).
Dziegielewski, 2013 [38]	Prospective	USA	81 patients with oropharyngeal cancer treated with transoral robotic surgery	N/A	HPV positive: 51 HPV negative: 20 p16 positive: 60 p16 negative: 11 Missing: 10	Stage I: 7 Stage III: 9 Stage IV: 63 Missing: 2 (AJCC 7th edition	Surgery: 81 Adjuvant RT: 69 Adjuvant CRT: 49	12 month post-operatively	HNCI	There was a significant change of overall attitude from baseline, but small clinically important difference and a good recovery at 12 months. No difference of overall attitude in HPV+ or HPV− patients (*p* = 0.56). Significant differences in overall attitude in patients who received adjuvant RT (*p* = 0.003) and those receiving adjuvant CRT (*p* = 0.04).
Goepfert, 2017 [40]	Cross-sectional	USA	935 patients diagnosed with oropharyngeal cancer between January 2000 and December 2014, aged 32–84 (median 56), 791 male, 144 female	N/A	Positive: 456 Negative: 59 Unknown: 420		RT alone: 276 CRT: 628 Surgery alone: 8 Surgery + CRT: 17 RT + salvage surgery: 6	1.5–15.6 years (median 6)	Decision regret scale, MDASI-HN	Patients reported a low level of decisional regret: mean score of 12.7/100 = “mild” 38.6% had no regret, 45.8% had “mild” regret, 15.5% of cohort had ”mod-strong” regret Regret significantly associated with higher T classification, combination treatment (surgery + RT/CRT), smoking at diagnosis, high MDASI-HN symptom score (associated with dysphagia symptom).
Janz, 2019 [28]	Prospective	USA	21 patients with HPV-associated oropharyngeal cancer, aged 49–76 (mean 58.2), 19 male, 2 female	17 patients with oral cavity cancer who smoke aged 32–76 (mean 55), 9 male, 8 female	Oropharynx cohort—Positive: 21/21 Oral cavity cohort—Positive 0/17	Stage IV (oropharynx): 16 Stage IV (oral cavity): 11 (AJCC 7th edition)	Surgery: 13 RT: 16 Chemotx: 17 **Combination therapiess:** Surgery + RT:2 CRT: 5 Surgery + CRT: 9 Other: 3	12 month follow-up	Assessment of Survivor Concerns instrument, CES-D, Cancer Behavior Inventory	At baseline, the HPV+ OPSCC patients had a mean cancer worry score of 2.8 and the oral cavity cohort had a score of 3.25 (*p* = 0.1) At baseline, the HPV+ OPSCC patients had a self-efficacy score of 97.8 and the oral cavity cohort had a scope of 96.3 (*p* = 0.79) Cancer worry decreased over time but was not statistically significant (2.8 to 2.4, *p* = 0.11)
Shaverdian, 2019 [39]	Retrospective	USA	24 consecutive patients enrolled in the CCRO-22 phase II clinical trial for locally advanced HPV-positive oropharyngeal cancer between March 2014 to March 2015, aged 49–83 (median 62), 21 male, 3 female.	N/A	Positive: 24	Stage III/IV: 24 (AJCC 7th edition)	Induction chemotherapy: 24 CRT: 24 (15 = 54 Gy, 10 = 60 Gy)	24 months (range of 16–30 months)	Decision Regret Scale, Chicago Priorities Scale	83% were “totally satisfied” with their treatment and its result. 17% said that they were “somewhat satisfied”. None had any level of dissatisfaction with the treatment. 92% “strongly agree” that their decision to proceed with de-escalated therapy was the “right decision”, 8% “agree”. 92% strongly disagreeing to the statement “I regret the choice I made”, none “agree” or “strongly agree”. 75% “strongly agree” with the statement “I would go for the same choice if I had to do it again”, 21% “agree” and the remaining 1 patient selected “neither agree nor disagree”. 92% “strongly agree” that their decision to receive de-escalated therapy was a “wise one”, with the remaining 8% patients selecting “agree”. The fear of disease recurrence was greater than expected in 42%, as expected in 33% and less than originally expected in 25%.
**Relationship and sexual behavior**										
Berg, 2021 [30]	Cross-sectional	Sweden	190 patients with BOT cancer, aged 33–84 (median 63), 137 male, 53 female	Patients with tonsillar cancer, general population	Positive: 131 Negative: 20 Missing: 39	Stage I–II: 27 Stage III–IV: 162 Missing: 1 (AJCC 7th edition)	RT: 56 CRT: 85 Surgery ± RT: 34 Surgery + CRT: 14 No adequate treatment: 1	15 months post-treatment	EORTC QLQ-C30, EORTC QLQ-H & N35	BOT cancer patients treated with radiotherapy alone reported worse sexuality scores than those treated with surgery and adjuvant CRT (40 versus 28). BOT cancer patients have worse (but not statistically significant) sexuality than the general population (*p* = 0.002), from tonsillar cancer patients (*p* = 0.16), comparing genders (*p* = 0.27), nor tumor stage (*p* = 0.44). HPV-negative patients report worse sexuality than HPV-positive patients (*p* = 0.05)
Casswell, 2021 [37]	Cross-sectional	Australia	136 patients with HPV-associated oropharyngeal cancer, aged 42–87 (median 61), 114 male, 22 female	N/A	Positive: 136/136	Stage I: 74 Stage II: 22 Stage III: 40 (AJCC 8th edition)	RT: 16 CRT: 120 Salvage surgery: 1	Mean 2.8 years post-treatment (range 1–5.5 years)	EORTC QLQ-C30, EORTC QLQ-SH22, MDASI-HN, PROMIS, Fear of Cancer Recurrence Inventory	An active sex life was considered important to the majority of survivors (60%) Only 20% of patients reported “quite a bit”/”very much” sexual activity in the 4 weeks prior Among those that reported high importance of an active sex life, 72% reported “little to no sexual activity” No difference in importance of sexual activity or recent sexual activity in patients who reported knowing if their cancer was caused by HPV Patients aware of the HPV association did not report negative changes more frequently in their general relationship (20% versus 7%), nor in their sexual relationship (39% versus 39%).
Taberna, 2017 [41]	Prospective	USA	172 patients with oropharyngeal cancer who self-reported that they were in a partnered relationship, aged 18–89, 125 male, 17 female (HPV+ cohort demographics)	90 patients with oral cavity cancer 81 partners of patients with oropharyngeal cancer	Positive: 142 Negative: 30	HPV+ cohort: Stage I: 5 Stage II: 7 Stage III: 43 Stage IV: 78 (AJCC 7th edition)	HPV+ cohort; Surgery: 45 CRT: 89 RT: 7 Chemo 1 Unknown 1	6-month follow up	Dyadic Adjustment Scale	Few patients or partners reported distressed relationships at baseline or at 6-months, with no significant difference when analyzed by HPV-status. Patients reported high relationship satisfaction; confided in their partner almost always (>85%), rarely/never regretted the relationship (~95%), and had high confidence in the latter (>75%). Strong majorities also described their relationships as happy/very happy (>90%). Demonstrations of affection: >65% agreed with their partner about sexual relations. The majority reported no issues in the relationship with regards to being too tired for sex (>65%) or not showing love (>80%). Very few patients reported relationship distress (T-score ≤ 40) in any subscale. 38% of HPV-positive patients reported that their relationship with their partner had not changed. When a change was perceived, it was generally positive, namely feeling supported by their partner (92%) and that their relationship had become stronger (69%). Approximately 25% of patients either blamed themselves for their cancer diagnosis (26%) or felt guilty about exposing their partner to HPV (28%).

#### 3.4.4. Relationship and Sexual Behavior

The relationship and sexual behavior domain comprised studies addressing impacts on sexuality and relationship quality/function.

Berg commented on sexuality in the context of a comparison of BOT cancer patients (n = 190) to patients with tonsillar carcinoma (n = 405) and to the general population (n = 190) [30]. Those treated with radiotherapy alone reported worse sexuality scores on the EORTC QLQ-HN35 than those who had surgery with adjuvant CRT (40 versus 28). Overall, BOT cancer patients and patients with HPV-negative disease reported worse sexuality scores than the general population and HPV-positive patients (36 versus 25, *p* = 0.002 and 48 versus 31, *p* = 0.05, respectively). There was no significant difference in subgroup analyses comparing subsite (BOT vs. tonsillar), gender, or disease stage.

Casswell utilized the EORTC Sexual Health Questionnaire (EORTC QLQ-SHQ-22) to assess the physical, social, and psychological aspects of sexual health [37]. This study demonstrated that an active sexual life is important to most in this cohort of HPV-associated OPSCC survivors (60%), but there was a much lower rate of recent significant sexual activity (20%). There was no difference in the rating of importance, nor in frequency of sexual activity in patients who knew their cancer was caused by HPV compared to those unaware of the viral etiology. The majority of patients reported no change (57%) or a positive change (27%) in the quality of relationships, while there was a negative impact on the sexual aspect of the relationship in 37% since their diagnosis.

Taberna’s prospective research compared the effects of diagnosis and treatment on relationship and sexual behavior in HPV-positive (n = 142) and negative patients (n = 120) [41]. In both groups, they found a high satisfaction with their relationship in elements such as honesty with their partner, lack of regret, confidence in the future of the partnership, and having an overall happy relationship, using the Dyadic Adjustment Scale. There was a significant decrease in frequency of sexual activity at 6-month follow-up for both cohorts (*p* < 0.01). 

### 3.5. Association of Psychosocial QOL and Treatment Modality

Berg reported no difference in depression, anxiety, or social quality of life scores when comparing OPSCC patients treated with primary CRT versus surgery with adjuvant RT/CRT [30]. Kaffenberger showed that patients receiving higher doses of RT to the ipsilateral parotid gland experienced higher anxiety levels and worse social function [27]. Goepfert’s study sub-analyzed decision regret based on treatment modality and found that receiving combined treatments (primary CRT or surgery and adjuvant RT/CRT) was an independent predictor of decisional regret [40]. Dziegielewski demonstrated significant differences in overall attitude in patients who received adjuvant RT (*p* = 0.003) and adjuvant CRT (*p* = 0.04) compared to those without adjuvant therapy [38].

### 3.6. Association of Psychosocial QOL and HPV Status

A summary of all HPV-related results is included in Table 3. Six studies did not have a non-HPV-associated OPSCC comparator group [27,33,35,36,37,39].

Six studies reported specific mental health and emotional wellbeing-related results for HPV-associated OPSCC patients. Berg and Korsten noted better emotional functioning in the HPV-positive cohort and Janz described a significant decrease in depression scores in the HPV-positive OPSCC cohort, without a significant decrease in the oral cavity cancer patients [28,30,33]. In the studies by Qualliotine, Rajeev-Kumar, and Shinn, no significant differences in mental health scores between HPV-positive versus HPV-negative cohorts were identified [29,31,34]. In the aforementioned studies, Qualliotine focused specifically on the mental health components of depression, Rajeev-Kumar on mood and anxiety, and Shinn on depression [29,31,34].

Berg reported better social functioning in the BOT OPSCC patients who were HPV-positive, using the EORTC QLQ-C30, while Dziegielewski did not find any difference in social function or social attitude between HPV-positive and negative cohorts, using the Head and Neck Cancer Inventory (HNCI) [30,38]. Korsten found that the HPV-positive cohort had worse social functioning during the treatment but recovered faster and to a greater degree in follow-up [32].

Dziegielewski found no difference in overall attitude based on HPV status, and Goepfert did not identify any difference in decisional regret in HPV-associated or non-associated patients [38,40]. There was a non-significant decrease of cancer worry at 12-month follow up for both the HPV-positive OPSCC patients and the oral cavity cancer patients described by Janz [28].

Taberna conducted the sole study describing the effect of HPV on relationship, finding no difference at baseline, but higher distress, though not significant, in the HPV-positive patients [41].

## 4. Discussion

This scoping review aimed to understand the landscape of published literature on the psychosocial QOL in oropharyngeal cancer patients and determine whether treatment regimens or HPV status play a role. Despite the rise in OPSCC globally and the current efforts to de-escalate treatments to allow for better QOL outcomes with long-term survival, this review identified a lack of observational research in this field.

The main mental health and emotional wellbeing theme findings are heterogeneous within the ten different studies. Interestingly, Janz, Berg, Qualliotine, and Korsten’s research all identified better mental health and emotional scores in patients with HPV-associated disease when compared to non-HPV-associated head and neck cancers (oropharyngeal or oral cavity) [28,30,31,32]. In contrast, Rajeev-Kumar and Shinn did not find an association with HPV status and mental health scores [29,34].

Within the social wellbeing and function domain, there were significant positive findings. Kaffenberger identified worse social scores in patients with higher RT doses to their ipsilateral parotid gland, and Dziegielewski found that post-operative social scores reached a low at three months but returned to baseline after one year [27,38]. Korsten noted that HPV-positive patients had better social functioning at baseline, worsened during treatment, but recovered to a greater level [32]. It is unclear why HPV-associated OPSCC patients have a greater toxicity burden from treatment, yet recover more quickly and better than HPV-negative patients; however, this is consistent with other research [42,43].

The stress category that emerged from the thematic analysis comprises four diverse concepts. These different feelings were identified in select subgroups within each study: younger patients and those with lower global QOL, higher symptom interference scores, and worse mental health scores had greater levels of fear of cancer recurrence [35,39]. A worse overall attitude/anxiety with function was found in patients receiving adjuvant therapy [38]. Greater decisional regret was reported in patients with higher T classification, those receiving combination treatment, smokers, and those with more dysphagia-related symptoms [39,40]. There was no difference in cancer worry scores between the groups compared (HPV-associated OPSCC patients vs. oral cavity cancer patients) [28].

The three studies focusing on relationship and sexual behavior employed three different PROMs, creating a challenge in comparison. Interestingly, none of the studies identified significant differences in their scores based on HPV status [30,37,41]. However, Taberna did learn that 28% of patients felt guilty about exposing their partner to HPV.

A secondary objective was to identify the impact of different treatment modalities on psychosocial QOL; however, only three studies addressed this topic. Kaffenberger compared advanced stage OPSCC patients treated for curative intent with CRT (non-surgical cohort) to surgery and adjuvant RT or CRT (surgical cohort) [27]. Social scores from the UW-QOL questionnaire and depression and anxiety-specific PROM screening did not demonstrate differences between the two treatments. Of note, patients who received higher doses of RT to the ipsilateral parotid gland experienced higher anxiety levels and worse social function. Goepfert’s study demonstrated a significant relationship between decisional regret scores and multimodality therapy [40]. While the cohort undergoing surgery and adjuvant therapy had the highest level of decisional regret, this represented few patients within the study and thus must be interpreted with caution (n = 17, 1.8% of study population). Shaverdian’s patients were enrolled in the CCRO-22 clinical trial and treated with neoadjuvant chemotherapy and de-escalated CRT based on individual response, reporting excellent decision regret outcomes [39]. The lack of a control group nonetheless limits analysis of psychosocial impact based on treatment modality.

With the rise in HPV-associated OPSCC, it is important to search for significant differences within the HPV-positive and HPV-negative cohorts. Higher emotional and social functioning and significant improvement in depression over time were found in the HPV-positive patients [28,30,32]. No significant differences were found in multiple other studies, and, specifically, no association of HPV status with any HNCI QOL domains [38], mood scores [29,31,34], decisional regret [40], or levels of relationship distress [41].

Within the thematic analysis, multiple PROMs were used for an individual symptom. It is clear that there is no standardized, uniform mental health survey specific for head and neck oncology patients, given that the ten studies within this review used eight different PROMs reporting on mental health or emotional well-being. The heterogeneity of PROMs utilized poses a difficulty in comparing outcomes. A recent study comparing different depression and anxiety PROMs in head and neck cancer patients found the prevalence of moderate and severe symptoms differed between surveys within the same patient cohort (using the Edmonton Symptom Assessment Scale, PHQ-9 and GAD-7) [44]. A meta-analysis performed by Krebber found that 8–24% of oncology patients suffered from depression, but these values were variable based on cancer type, treatment phase, and the screening instrument used to measure depression [45]. Similarly, one of the studies included in this review reported a significant association of depression and overall survival in multivariable modelling using the PHQ-9, while there was no significant risk for mortality using the CES-D [34]. While PROMs are valuable, establishing standard, agreed-upon metrics tailored to the head and neck patient population will be an important future goal to create comparable research outcomes and to decrease survey fatigue [46].

Many completed or ongoing clinical trials are attempting to change the standard treatments for HPV-positive OPSCC and reduce the treatment-related secondary effects [4,12,13]. Patients can be offered a plethora of potential treatments. This era of patient-centered decision making may open the door for further distress due to a shift of responsibility to the patient and the possibility of decision regret [47]. Windon performed a qualitative analysis of treatment decision-making in OPSCC patients who were offered surgery or RT as primary curative intent treatments [48]. Challenges in decision making included the difficulty of incorporating the perceived recommendation of the physician, personal desire for tumor excision, fear of specific secondary effects of treatment, and individual values.

In this scoping review, decision regret was measured in an HPV-positive OPSCC cohort enrolled in a de-escalation clinical trial [39]. At 16 to 30 months post-treatment, patients logged excellent scores on the Decision Regret Scale. In general, late RT-related adverse events, commonly xerostomia and dysphagia, are often the main drivers in post-treatment negative QOL [49,50,51,52]. The goal of lowered RT doses is shared in other trials to minimize these secondary effects [53,54,55]. While Shaverdian’s results are positive, it is important to note that this was a small population (n = 24) and a median two-year follow-up may not have provided adequate time to capture the late post-radiation adverse effects. Goepfert assessed a large OPSCC cohort (n = 935) at a median of six years post-treatment, noting mild levels of decisional regret on average [40]. Higher levels of decision regret were associated with higher T staging, multimodal treatment, smoking at diagnosis, and high MDASI-HN symptom score. Decision regret is not yet well studied in OPSCC, despite current efforts to change the standard-of-care treatment, and this is a potential outcome to consider in future research.

There are several limitations to note within this scoping review. While an extensive search of four large databases with diverse target audiences was performed, additional databases may have yielded further results. Grey literature was not explored and published abstracts were not included. This was decided because the lack of full available data would not allow for analysis. Finally, the heterogeneity of themes and patient-reported outcome measures limited the ability to compare studies and draw conclusions.

## 5. Conclusions

This review has reported the current status of emotional, social, and psychological QOL in OPSCC survivors. With the rise of HPV-related OPSCC and characteristically younger, healthier patients with improved prognostication, treatment-related morbidity and associated psychosocial impact is now a key area of discussion amongst advocacy groups and oncology professionals alike. Specifically, decisional regret within the category of stress and the impact on relationships and sexuality have been recognized as unique avenues for future research, given the many ongoing clinical trials and the association of OPSCC with HPV, respectively. Few studies have explored these concepts, and no review has focused on these outcomes thus far. This scoping review identified a need to establish a uniform head and neck oncology-specific QOL metric to more consistently assess psychosocial burden within these patients.

## Figures and Tables

**Table 1:** Inclusion and exclusion criteria.

Abstract Criteria		
Study Characteristics	Inclusion Criteria	Exclusion Criteria
Participants (population)	- Adults, aged 18+ - Oropharyngeal squamous cell carcinoma diagnosis	- Other cancers (head and neck carcinomas or otherwise, if oropharyngeal squamous cell carcinoma is not specified for)
Study design (concept)	- Observational studies with a psychosocial focus	- Secondary research - Published guidelines - Cost-effectiveness studies
Outcome measures (context)	- Validated patient-reported outcome measures with specific mention of psychosocial quality of life	- Quality of life not reported in the abstract - Qualitative research without validated metrics
Other (publication)	- Published in a peer-reviewed journal - English language	- Dissertations/thesis - Study protocols - Conference proceedings - Non-English language
**Full-text criteria (additional criteria)**		
Study design	- HPV status testing performed	
Outcome measures	- Psychosocial quality of life is an outcome of the study	- Psychosocial quality of life is not a focus of the study

**Table 3:** Summary of results comparing HPV-positive and HPV-negative cohorts. Bolded = significant results.

Study	HPV-Related Results
**Mental Health and Emotional Wellbeing**	
Berg, 2021 [30]	**HPV-positive BOT cancer patients had better emotional functioning (*p* = 0.004) than the HPV-negative cohort on EORTCQLQ-C30** (Primary text, Table 5)
Janz, 2019 [28]	At baseline, HPV-positive OPSCC cohort had a non-significant difference in mean depression score compared to smoking oral cavity patients (12 versus 14, *p* = 0.41).
	**Depression decreased significantly over time for the HPV-positive OPSCC patients (12 to 9.9, *p* = 0.03) and non-significantly in the oral cavity patients (14 to 9.73, *p* = 0.1) from baseline to 12 months.**
Korsten, 2021 [32]	**Emotional functioning was significantly different between HPV-positive and negative patients: average scores were equal at baseline and in close follow-up (6 weeks and 3 months), but scores improved more in HPV-positive patients (*p* = 0.039).**
Qualliotine, 2017 [31]	There was no significant association noted between depression and HPV status (*p* > 0.1) (Primary text: Figure 1).
Rajeev-Kumar, 2019 [29]	There is no statistically significant relationship between anxiety or mood and human papillomavirus infection status (*p* = 0.089 for anxiety; *p* = 0.731 for mood).
Shinn, 2016 [34]	There was no significant difference in depression scores between HPV-positive and HPV-negative patients.
**Social wellbeing and function**	
Berg, 2021 [30]	**HPV-positive BOT cancer patients had better social functioning (*p* = 0.01) than the HPV-negative cohort on EORTCQLQ-C30** (Primary text, Table 5)
Dziegielewski, 2013 [38]	HPV status did not correlate with any quality of life domain (i.e., social function, social attitude, overall attitude) in the HNCI (*p* > 0.5 for all domains, Primary text: Table 5)
Korsten, 2021 [32]	**Social functioning recovered faster and to a better degree in HPV-positive patients (*p* = 0.033)** (Primary text: Figure 2).
**Stress**	
Goepfert, 2017 [40]	There was no significant difference in MDASI-HN symptom scores (*p* = 0.27) or proportional decisional regret (*p* = 0.37) based on HPV status (Primary text: Table 3)
Janz, 2019 [28]	At baseline, HPV-positive OPSCC cohort had a non-significant difference in mean cancer worry compared to smoking oral cavity patients (2.8 versus 3.25, *p* = 0.1).
	Cancer worry decreased non-significantly over time in both the HPV-positive OPSCC patients (2.8 to 2.4, *p* = 0.11) and the oral cavity patients (3.2 to 2.7, *p* = 0.07). (Primary text: Table 2)
**Relationship and sexual behavior**	
Taberna, 2017 [41]	At baseline, there was no statistically significant differences in levels of relationship distress between HPV-positive and HPV-negative patients. At 6 months follow up, a non-significant trend was noted of higher distress in the affection expression subscale of the DAS for HPV-positive patients compared to HPV-negative. 38% of HPV-positive patients reported that their relationship with their partner had stayed the same, and those who reported a change felt it was positive. 70% of partners reported favorable changes in their relationship since diagnosis. **A higher proportion of partners reported more stress in their relationship since the cancer diagnosis than the patients (39% versus 14%, *p* < 0.01).** Approximately a quarter of patients blamed themselves for their cancer diagnosis or felt guilty about exposing their partner to HPV. 14% of partners felt guilty for possibly exposing their partner to HPV or were concerned that the HPV infection may have been a result of an extramarital relationship (their or their partner’s). **There was a significant decline in sexual behavior frequency in both HPV-positive and HPV-negative cohorts** (Primary text: Figure 2, *p* < 0.01).
**No comparison**	
Casswell, 2021 [35]	N/A
Casswell, 2021 [37]	N/A
Kaffenberger, 2021 [27]	N/A
Lee, 2022 [33]	N/A
McDowell, 2021 [36]	N/A
Shaverdian, 2019 [39]	N/A

N/A—Not available, study does not have an HPV-negative comparator group.

## Data Availability

The datasets used and/or analyzed during the current study are available from the corresponding author on reasonable request.

## Supplementary Materials

The following supporting information can be downloaded at: https://www.mdpi.com/article/10.3390/jcm12062122/s1.
