# Supplementary material for: Patient-Reported Outcome Measures of Psychosocial Quality of Life in Oropharyngeal Cancer Patients: A Scoping Review
Source: J Clin Med. 2023 Mar 8;12(6):2122. doi: 10.3390/jcm12062122 (PMC10057395; doi:10.3390/jcm12062122)
Supplement: Supplementary file 1 [file jcm-12-02122-s001.zip › jcm-2164143-supplementary.pdf]

Supplementary S1: Scoping review database search

Medline
exp Oropharyngeal Neoplasms/
Pharyngeal Neoplasms/
limit 2 to yr="1966-1984"
(Oropharyn* adj3 (carcinoma* or cancer* or lesion* or malignan* or tumor* or tumour* or neoplasm*)).ti,ab,kf.
1 or 3 or 4
"Quality of Life"/
depression/
exp Emotions/
depressive disorder/
depressive disorder, major/
psychosocial functioning/
Psychology, Developmental/
limit 12 to yr="2015 - 2020"
"quality of life".ti,ab,kf.
(well being or wellbeing).ti,ab,kf.
(depressi* or depressed or anxiety or anxious or sadness or sad or happiness or happy or anger or angry or afraid or fear or agoraphobi* or stress or stressful or stressor* or panic* or burnout or burn out or isolation).ti,ab,kf.
6 or 7 or 8 or 9 or 10 or 11 or 13 or 14 or 15 or 16
5 and 17
Affective Symptoms/
Mental Health/
Life Change Events/
Dysthymic Disorder/
Anxiety Disorders/
Agoraphobia/
Panic Disorder/
Stress, Psychological/
Burnout, Psychological/
Adjustment Disorders/
((emotion* or mental* or psychologic* or social* or psychosocial* or psycho social*) adj2 (health or stability or stable)).ti,ab,kf.
or/19-29
17 or 30
5 and 31
patient reported outcome measures/
Patient Outcome Assessment/
limit 34 to yr="2013 - 2016"
"Activities of Daily Living"/

(patient reported outcome* or Patient Centered Outcome* or Patient Centred Outcome*).ti,ab,kf.
30 or 33 or 35 or 36 or 37
17 or 38
5 and 39
6 or 7 or 8 or 9 or 10 or 11 or 13 or 14 or 15 or 16 or 19 or 20 or 21 or 22 or 23 or 24 or 25 or 26 or 27 or 28 or 29 or 33 or 35 or 36 or 37
5 and 41

Embase
exp oropharynx tumor/
(Oropharyn* adj3 (carcinoma* or cancer* or lesion* or malignan* or tumor* or tumour* or neoplasm*)).ti,ab,kw
"quality of life"/
depression/
exp emotion/
major depression/
social psychology/
developmental psychology/
limit 8 to yr="2007 -Current"
"quality of life".ti,ab,kw.
(well being or wellbeing).ti,ab,kw.
(depressi* or depressed or anxiety or anxious or sadness or sad or happiness or happy or anger or angry or afraid or fear or agoraphobi* or stress or stressful or stressor* or panic* or burnout or burn out or isolation).ti,ab,kw.
emotional disorder/
mental health/
life event/
dysthymia/
anxiety disorder/
agoraphobia/
panic/
mental stress/
burnout/
adjustment disorder
((emotion* or mental* or psychologic* or social* or psychosocial* or psycho social*) adj2 (health or stability or stable)).ti,ab,kw.
patient-reported outcome/
daily life activity/
(patient reported outcome* or Patient Centered Outcome* or Patient Centred Outcome*).ti,ab,kw.

CINAHL
(MH "Oropharyngeal Neoplasms+")

TI (oropharyn* N2 (carcinoma* or cancer* or lesion* or malignan* or tumor* or tumour* or neoplasm*)) OR AB (oropharyn* N2 (carcinoma* or cancer* or lesion* or malignan* or tumor* or tumour* or neoplasm*))
S1 OR S3
(MH "Quality of Life")
(MH "Depression")
(MH "Emotions+")
(MH "Psychosocial Functioning")
TI quality of life OR AB quality of life
TI (well being or wellbeing or well-being) OR AB (well being or wellbeing or well-being)
TI ((depressi* or depressed or anxiety or anxious or sadness or sad or happiness or happy or anger or angry or afraid or fear or agoraphobi* or stress or stressful or stressor* or panic* or burnout or burn out)) AND AB ((depressi* or depressed or anxiety or anxious or sadness or sad or happiness or happy or anger or angry or afraid or fear or agoraphobi* or stress or stressful or stressor* or panic* or burnout or burn out))
(depressi* or depressed or anxiety or anxious or sadness or sad or happiness or happy or anger or angry or afraid or fear or agoraphobi* or stress or stressful or stressor* or panic* or burnout or burn out or isolation).ti,ab,kw.
(MH "Mental Health")
(MH "Life Change Events")
(MH "Dysthymic Disorder")
(MH "Anxiety Disorders")
(MH "Agoraphobia")
(MH "Panic Disorder")
(MH "Stress, Psychological")
(MH "Adjustment Disorders")
TI (((emotion* or mental* or psychologic* or social* or psychosocial*) N2 (health or stability or stable))) OR AB (((emotion* or mental* or psychologic* or social* or psychosocial*) N2 (health or stability or stable)))
S5 OR S6 OR S7 OR S8 OR S9 OR S10 OR S11 OR S12 OR S13 OR S14 OR S15 OR S16 OR S17 OR S18 OR S19 OR S20 OR S21 OR S22
S4 AND S23
(MH "Patient-Reported Outcomes")
(MH "Activities of Daily Living")
TI ((patient reported outcome* or Patient Centered Outcome* or Patient Centred Outcome*)) OR AB ((patient reported outcome* or Patient Centered Outcome* or Patient Centred Outcome*))

PsychInfo
(Oropharyn* adj2 (carcinoma* or cancer* or lesion* or malignan* or tumor* or tumour* or neoplasm*)).mp.
"Quality of Life"/
"Depression (Emotion)"/ or Major Depression/ or Reactive Depression/
exp Emotions/

"quality of life".mp.
(well being or wellbeing).mp.
(depressi* or depressed or anxiety or anxious or sadness or sad or happiness or happy or anger or angry or afraid or fear or agoraphobi* or stress or stressful or stressor* or panic* or burnout or burn out).mp.
Affective Disorders/
Mental Health/
Life Changes/
Dysthymic Disorder/
Anxiety Disorders/
Agoraphobia/
Panic Disorder/
Psychological Stress/
Adjustment Disorders/
((emotion* or mental* or psychologic* or social* or psychosocial* or psycho social*) adj2 (health or stability or stable)).mp.
Patient Reported Outcome Measures/
(patient reported outcome* or Patient Centered Outcome* or Patient Centred Outcome*).mp.

Supplementary S2: Frequency of quality of life measures

Quality of Life Measure	Frequency used	Author, Year
General / Head and Neck		
**The European Organisation of Research and Treatment of Cancer Quality of Life Questionnaire (EORTC QLQ-C30)**[1]<br><br>This 30-item questionnaire incorporates nine multi-item scales: five functional scales (physical, role, cognitive, emotional, and social); three symptom scales (fatigue, pain, and nausea and vomiting); and a global health and quality-of-life scale.	4	Berg, 2021[2]<br>Casswell, 2021[3]<br>Casswell, 2021[4]<br>McDowell, 2021[5]<br>Korsten, 2021[6]
**The European Organisation of Research and Treatment of Cancer Head and Neck Cancer module (EORTC QLQ-H&N35)**[7]<br><br>This 35-item questionnaire is a site-specific module to be utilized with the core questionnaire. Specifically, it consists of seven multi-item scales (oral pain, problems with swallowing, senses, speech, social eating and social contact) and 11 single-item scales, assessing problems with teeth, mouth opening, dry mouth, sticky saliva, coughing, feeling ill, as well as use of analgesics, nutritional supplements, feeding tube and weight change (gain or loss)..	2	Berg, 2021[2]<br>McDowell, 2021[5]<br>Korsten, 2021[6]
**The University of Washington Quality of Life Questionnaire (UW-QOL)**[8,9]<br><br>This questionnaire was one of the first head and neck specific quality of life metrics published in 1993. The latest version has 12 domains, including pain, appearance, activity, recreation, swallowing, chewing, speech, shoulder, taste, saliva, mood, and anxiety. Each is scored along a range from no symptoms to severe symptoms associated with that domain.	3	Kaffenberger, 2021[10]<br>Rajeev-Kumar, 2019[11]<br>Shaverdian, 2019[12]<br>Lee, 2022[13]
**The MD Anderson Symptom Inventory for Head and Neck Cancer (MDASI-HN)**[14]<br><br>The 28-item MDASI-HN module is designed to assess the burden and severity of symptoms and the interference these have on daily functioning. It includes 13 general cancer and 9 HNC-specific symptoms; an additional 6 items assess the interference of symptoms in daily activities. Reported scores include a mean symptom severity score of all items (22 symptom items) and a mean symptom interference score (6 interference items).	5	Casswell, 2021[3]<br>Casswell, 2021[4]<br>Goepfert, 2017[15]<br>Janz, 2019[16]<br>McDowell, 2021[5]
**The Head and Neck Cancer Inventory (HNCI)**[15]<br><br>This 30-item questionnaire is a multidimensional head and neck cancer specific survey assessing four domains: speech, eating, social disruption, and aesthetics. Each domain has two components, the functional ability to perform the task and the attitudinal satisfaction with the task.	1	Dziegielewski, 2013[17]
Mental Health		
**Centers for Epidemiological Studies-Depression Scale (CES-D)**[18]<br><br>This is a 20 items questionnaire (or 10 item short survey) that assesses mood, somatic complaints, interactions with others, and motor functioning. The response values are 4-point Likert scales, with range 0-3, indicating how frequently in the previous week the patient experienced each symptom.	2	Qualliotine, 2017[19]<br>Shinn, 2016[20]
**Physicians Health Questionnaire (PHQ-9)**[21]<br><br>This questionnaire assesses the 9 Diagnostic and Statistical Manual IV (DSM-IV) criteria for major depressive episode. This can be used as a self-administered screening tool for depression.	2	Kaffenberger, 2021[10]<br>Shinn, 2016[20]
**Generalized anxiety disorder (GAD-7)**[22,23]<br><br>This 7-item anxiety scale based on DSM-IV (Text Revision) criteria for generalized anxiety disorder. This can be used as a self-administered screening tool for anxiety.	1	Kaffenberger, 2021[10]
**Patient-Reported Outcomes Measurement Information System (PROMIS®)**[24]<br><br>These questionnaires are created and sponsored by the National Institutes of Health's (NIH) and there are several short-form patient-reported outcome measures that target specific symptoms. Specifically, the Emotional Distress-Anxiety 7a, (7 items) and Emotional Distress-Depression 8b (8 items) were utilized.	3	Casswell, 2021[3]<br>Casswell, 2021[4]<br>McDowell, 2021[5]
Stress		
**Fear of Cancer Recurrence Inventory (FCRI)**[25]	1	Casswell, 2021[3]

The 9-item questionnaire assesses the presence, frequency, intensity, and duration of thoughts associated with fear of cancer recurrence over the preceding month. The short-form is an adapted metric utilizing the severity domain and it was validated to provide a cut-off value for screening for clinical levels of fear of cancer recurrence.		
**Assessment of Survivor Concerns instrument**[26]<br><br>This 5-item questionnaire is composed of three elements related to cancer worry, and two applying to general health worry. This survey can be used when an assessment of cancer survivor concerns is needed and is appropriate in both short-term and long-term survivor populations.	1	Janz, 2019[16]
**Cancer Behavior Inventory**[27]<br><br>This 14-item questionnaire is a shortened version of the same named survey. It incorporates two items from each of the following domains: maintenance of activity and independence, coping with treatment-related side effects, accepting cancer/maintaining positive attitude, seeking and understanding medical information, affective regulation, seeking support, and stress management for medical appointments.	1	Janz, 2019[16]
**Decision Regret Scale**[28]<br><br>This 5-item questionnaire measures distress or remorse after a healthcare-related decision. Patients select along a range of strongly agree to strongly disagree when reading the statements.	2	Goepfert, 2017[15]<br>Shaverdian, 2019[12]
**Patients Concerns Inventory (PCI)**[29]<br><br>This 45-item questionnaire is used to identify what topics a patient would like to discuss during their consultation. The PCI is broad providing many options for patients to select, including hearing, intimacy, fatigue, financial/benefits, PEG tube, relationships, regret, support for family, and wound healing. This scale allows personalization of patient consultations.	1	Shaverdian, 2019[12]
Relationship		
**The European Organisation of Research and Treatment of Cancer Sexual Health Questionnaire (EORTC QLQ-SHQ-22)**[30]<br><br>This 22-item questionnaire explores sexual health in a multidimensional manner, incorporating physical, psychological, and social aspects. It includes an 8-item sexual satisfaction scale, a 3-item sexual pain scale, and 11 single items which are either functional or symptom scales).	1	Casswell, 2021[4]
**Dyadic Adjustment Scale**[31]<br><br>This 32-item questionnaire initially developed in 1976 to assess the quality of marriage and other such dyads. It incorporates the subscales of satisfaction, cohesion, consensus and affectional expression.	1	Taberna, 2017[32]
Other		
**Eating Assessment Tool-10 (EAT-10)**[33]<br><br>The EAT-10 10-item survey is used for the subjective assessment of dysphagia and has been developed to document initial severity of patient's dysphagia and to monitor response to treatment in patients with swallowing disorders.	1	Kaffenberger, 2021[10]
**Neck Disability Index (NDI)**[34]<br><br>This 10-item scaled questionnaire was developed to assess the impact of function of activities of daily living due to neck pain. This survey items include pain intensity, personal care, lifting, sleeping, driving, recreation, headaches, concentration, reading and work – all factors that can lead to chronic disability.	1	Kaffenberger, 2021[10]

References:

1. Aaronson NK, Ahmedzai S, Bergman B, et al. The European Organization for Research and Treatment of Cancer QLQ-C30: a quality-of-life instrument for use in international clinical trials in oncology. *J Natl Cancer Inst.* 1993;85(5):365-376.
2. Berg M, Adnan A, Hogmo A, et al. A national study of health-related quality of life in patients with cancer of the base of the tongue compared to the general population and to patients with tonsillar carcinoma. *Head and Neck.* 2021.
3. Casswell G, Gough K, Drosdowsky A, et al. Fear of Cancer Recurrence in Survivors of Human Papillomavirus-Associated Oropharyngeal Carcinoma. *International Journal of Radiation Oncology, Biology, Physics.* 2021;13:13.
4. Casswell G, Gough K, Drosdowsky A, et al. Sexual Health and Interpersonal Relationships After Chemoradiation Therapy for Human Papillomavirus-Associated Oropharyngeal Cancer: A Cross-sectional Study. *International Journal of Radiation Oncology, Biology, Physics.* 2021;110(2):382-393.
5. McDowell L, Casswell G, Bressel M, et al. Symptom burden, quality of life, functioning and emotional distress in survivors of human papillomavirus associated oropharyngeal cancer: An Australian cohort. *Oral Oncology.* 2021;122.
6. Korsten LHA, Jansen F, Lissenberg-Witte BI, et al. The course of health-related quality of life from diagnosis to two years follow-up in patients with oropharyngeal cancer: does HPV status matter? *Supportive Care in Cancer.* 2021;29(8):4473-4483.
7. Bjordal K, de Graeff A, Fayers PM, et al. A 12 country field study of the EORTC QLQ-C30 (version 3.0) and the head and neck cancer specific module (EORTC QLQ-H&N35) in head and neck patients. EORTC Quality of Life Group. *Eur J Cancer.* 2000;36(14):1796-1807.
8. Hassan SJ, Weymuller EA, Jr. Assessment of quality of life in head and neck cancer patients. *Head Neck.* 1993;15(6):485-496.
9. Rogers SN, Gwanne S, Lowe D, Humphris G, Yueh B, Weymuller EA, Jr. The addition of mood and anxiety domains to the University of Washington quality of life scale. *Head Neck.* 2002;24(6):521-529.
10. Kaffenberger TM, Patel AK, Lyu L, et al. Quality of life after radiation and transoral robotic surgery in advanced oropharyngeal cancer. *Laryngoscope Investigative Otolaryngology.* 2021.
11. Rajeev-Kumar G, Moreno J, Kelley A, Sharma S, Gupta V, Bakst R. Emotional Quality of Life After Radiation Therapy for Oropharyngeal Carcinoma. *Advances in radiation oncology.* 2019;4(4):674-682.
12. Shaverdian N, Hegde JV, Felix C, et al. Patient perspectives and treatment regret after de-escalated chemoradiation for human papillomavirus-positive oropharyngeal cancer: Findings from a phase II trial. *Head & Neck.* 2019;41(8):2768-2776.
13. Lee E, Crowder HR, Gorelik D, et al. Comparison of quality of life outcomes in a de-intensification treatment regimen for p16 + oropharyngeal cancer. *Eur Arch Otorhinolaryngol.* 2022;279(9):4533-4540.
14. Rosenthal DI, Mendoza TR, Chambers MS, et al. Measuring head and neck cancer symptom burden: the development and validation of the M. D. Anderson symptom inventory, head and neck module. *Head Neck.* 2007;29(10):923-931.
15. Goepfert RP, Fuller CD, Gunn GB, et al. Symptom burden as a driver of decisional regret in long-term oropharyngeal carcinoma survivors. *Head & Neck.* 2017;39(11):2151-2158.
16. Janz TA, Momin SR, Sterba KR, Kato MG, Armeson KE, Day TA. Comparison of psychosocial factors over time among HPV+ oropharyngeal cancer and tobacco-related oral cavity cancer patients. *American Journal of Otolaryngology.* 2019;40(1):40-45.

17. Dziegielewski PT, Teknos TN, Durmus K, et al. Transoral robotic surgery for oropharyngeal cancer: long-term quality of life and functional outcomes. *JAMA Otolaryngology-- Head & Neck Surgery.* 2013;139(11):1099-1108.
18. Irwin M, Artin KH, Oxman MN. Screening for depression in the older adult: criterion validity of the 10-item Center for Epidemiological Studies Depression Scale (CES-D). *Arch Intern Med.* 1999;159(15):1701-1704.
19. Qualliotine JR, Califano JA, Li RJ, et al. Human papillomavirus tumour status is not associated with a positive depression screen for patients with oropharyngeal cancer. *Journal of Laryngology & Otology.* 2017;131(9):760-767.
20. Shinn EH, Valentine A, Jethanandani A, et al. Depression and Oropharynx Cancer Outcome. *Psychosomatic Medicine.* 2016;78(1):38-48.
21. Spitzer RL, Kroenke K, Williams JB. Validation and utility of a self-report version of PRIME-MD: the PHQ primary care study. Primary Care Evaluation of Mental Disorders. Patient Health Questionnaire. *Jama.* 1999;282(18):1737-1744.
22. Spitzer RL, Kroenke K, Williams JB, Löwe B. A brief measure for assessing generalized anxiety disorder: the GAD-7. *Arch Intern Med.* 2006;166(10):1092-1097.
23. Segal DL. Diagnostic and Statistical Manual of Mental Disorders (DSM-IV-TR). In: *The Corsini Encyclopedia of Psychology.*1-3.
24. Pilkonis PA, Choi SW, Reise SP, Stover AM, Riley WT, Cella D. Item banks for measuring emotional distress from the Patient-Reported Outcomes Measurement Information System (PROMIS®): depression, anxiety, and anger. *Assessment.* 2011;18(3):263-283.
25. Simard S, Savard J. Screening and comorbidity of clinical levels of fear of cancer recurrence. *J Cancer Surviv.* 2015;9(3):481-491.
26. Gotay CC, Pagano IS. Assessment of Survivor Concerns (ASC): a newly proposed brief questionnaire. *Health Qual Life Outcomes.* 2007;5:15.
27. Merluzzi TV, Martinez Sanchez MA. Assessment of self-efficacy and coping with cancer: development and validation of the cancer behavior inventory. *Health Psychol.* 1997;16(2):163-170.
28. Brehaut JC, O'Connor AM, Wood TJ, et al. Validation of a decision regret scale. *Med Decis Making.* 2003;23(4):281-292.
29. Rogers SN, El-Sheikha J, Lowe D. The development of a Patients Concerns Inventory (PCI) to help reveal patients concerns in the head and neck clinic. *Oral Oncol.* 2009;45(7):555-561.
30. Oberguggenberger AS, Nagele E, Inwald EC, et al. Phase 1-3 of the cross-cultural development of an EORTC questionnaire for the assessment of sexual health in cancer patients: the EORTC SHQ-22. *Cancer Med.* 2018;7(3):635-645.
31. Spanier GB. Measuring Dyadic Adjustment: New Scales for Assessing the Quality of Marriage and Similar Dyads. *Journal of Marriage and Family.* 1976;38(1):15-28.
32. Taberna M, Inglehart RC, Pickard RK, et al. Significant changes in sexual behavior after a diagnosis of human papillomavirus-positive and human papillomavirus-negative oral cancer. *Cancer.* 2017;123(7):1156-1165.
33. Belafsky PC, Mouadeb DA, Rees CJ, et al. Validity and reliability of the Eating Assessment Tool (EAT-10). *Ann Otol Rhinol Laryngol.* 2008;117(12):919-924.
34. Vernon H, Mior S. The Neck Disability Index: a study of reliability and validity. *J Manipulative Physiol Ther.* 1991;14(7):409-415.
